# Urbanisation, risk stratification and house infestation with a major vector of Chagas disease in an endemic municipality of the Argentine Chaco

**DOI:** 10.1186/s13071-020-04182-3

**Published:** 2020-06-18

**Authors:** María Sol Gaspe, María del Pilar Fernández, Marta Victoria Cardinal, Gustavo Fabián Enriquez, Lucía Inés Rodríguez-Planes, Natalia Paula Macchiaverna, Ricardo Esteban Gürtler

**Affiliations:** 1 Laboratorio de Eco-Epidemiología, Facultad de Ciencias Exactas y Naturales, Universidad de Buenos Aires, Ciudad Universitaria, C1428EHA Buenos Aires, Argentina; 2 Instituto de Ecología, Genética y Evolución de Buenos Aires, Consejo Nacional de Investigaciones Científicas y Técnicas, Ciudad Universitaria, C1428EHA Buenos Aires, Argentina; 3 Earth Institute, Columbia University, New York, NY 10025 USA; 4 Instituto de Ciencias Polares, Ambiente y Recursos Naturales, Universidad Nacional de Tierra del Fuego, Onas 450, 9410 Ushuaia, Argentina

**Keywords:** Gran Chaco, Rural, *Triatoma infestans*, Urban, Vector control

## Abstract

**Background:**

The occurrence of the major vectors of Chagas disease has historically been linked to poor rural housing, but urban or peri-urban infestations are increasingly being reported. We evaluated a simple risk index to detect houses infested with *Triatoma infestans* and tested whether house infestation and vector abundance increased across the urban-to-rural gradient in Avia Terai, an endemic municipality of the Argentine Chaco; whether the association between infestation and selected ecological determinants varied across the gradient; and whether urban and peri-urban infestations were associated with population settlement history.

**Methods:**

We conducted a screening survey of house infestation in 2296 urban, peri-urban and rural dwellings to identify high-risk houses based on a simple index, and then searched for triatomines in all high-risk houses and in a systematic sample of low-risk houses.

**Results:**

The risk index had maximum sensitivity and negative predictive value, and low specificity. The combined number of infested houses in peri-urban and urban areas equalled that in rural areas. House infestation prevalence was 4.5%, 22.7% and 42.4% across the gradient, and paralleled the increasing trend in the frequency of domestic animals and peridomestic structures. Multiple logistic regression analysis showed that house infestation was positively and significantly associated with the availability of poultry and bug refuges in walls, and was negatively associated with domestic insecticide use. Several pieces of evidence, including absence of spatial aggregation of house infestation, support that *T. infestans* has been a long-established occupant of urban, peri-urban and rural settings in Avia Terai.

**Conclusions:**

An integrated vector management strategy targeting chicken coops and good husbandry practices may provide more cost-effective returns to insecticide-based vector elimination efforts.
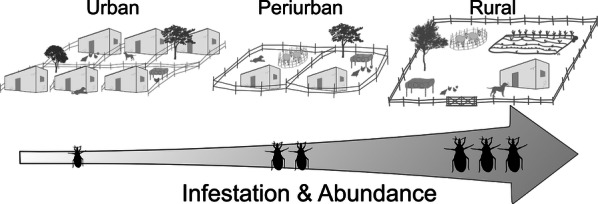

## Background

The human population became increasingly urban during the 20th century. The global urbanisation trend is expected to continue during the next decades [1] and has major implications for human health, particularly in low- and middle-income countries where unplanned urbanisation has led to impoverished settlements with increased transmission of infectious diseases [2]. Most of the regions undergoing fast urbanisation overlap with high-risk areas of neglected tropical diseases (NTDs) [3]. This changing environment led to the (re)emergence of many NTDs which may become more intense in the near future [4]. The changing epidemiology of NTDs in these regions presents new challenges to health policies and to the achievement of Sustainable Developmental Goals [3]. Multidisciplinary research on transmission risk factors in urban settings is essential for evidence-based policy development and decision making [5].

Chagas disease is among the most important NTDs in Latin America, where approximately 6 million people are infected with *Trypanosoma cruzi*, its etiological agent [6]. More than 80% of the population of Latin American countries lived in cities by the year 2018 [1]. Urbanisation added new features to the eco-epidemiology of Chagas disease or modified the existing ones [7]. Traditionally associated with poor rural environments, Chagas disease had expanded into peri-urban and urban centers [8–10], and into non-endemic areas through international migration [11]. The transmission of *T. cruzi* is mainly mediated by triatomine bugs, but also occurs from mother-to-newborn, through blood transfusion or organ transplant, and by ingestion of contaminated foodstuff.

Urban transmission of arthropod-mediated pathogens is increasing [12]. At least 14 triatomine species naturally infected with *T. cruzi* invade and colonise peri-urban and urban dwellings in endemic areas [13]. Relevant examples include *Triatoma infestans*, *T. dimidiata*, *T. pallidipennis*, *T. tibiamaculata*, *Panstrongylus geniculatus* and *Rhodnius neglectus*, among others (e.g. [9, 14–19]). *Triatoma infestans*, the main vector of *T. cruzi* in the Gran Chaco region, has increasingly being reported to colonise houses in peri-urban and urban areas of Argentina, Bolivia and Peru, and established local transmission cycles [9, 10, 20–23]. Whether urban infestation is a (re)emerging threat or a long-standing, overlooked issue is unclear and has implications for a mechanistic understanding of system dynamics and policy development. Investigation of the patterns of house infestation in urban or peri-urban areas and their putative links to rural infestations (e.g. [24]) may cast light on the underlying processes and assist in the design of appropriate vector control strategies.

The main ecological determinants of house infestation with *T. infestans* in rural settings are domestic host availability, appropriate refuges for triatomines (i.e. building materials), and household vector control practices [14, 25–28]. Similarly, in urban and peri-urban communities, the presence of guinea pigs or chickens, the number of human residents per room and building materials were all positively associated with house infestation with *T. infestans* [9, 10, 22, 23]. Migration and settlement features also affect house infestation and parasite transmission in peri-urban and rural environments [22, 24, 29, 30]. To our knowledge, a detailed analysis of house infestation patterns across the urban-to-rural gradient of a Chagas disease-endemic district has not been performed yet, nor have its determinants been assessed comparatively. A rapid identification of high-risk houses in populated peri-urban or urban settings would bring enormous savings in labour and other resources.

This study was conceived as part of a rapid intervention package to suppress house infestation with *T. infestans* and reduce the disease burden in Avia Terai municipality in a sustainable manner. Interventions were articulated with the health and education sectors both at local and provincial levels at the outset, and included an educational intervention in order to mobilise and engage local communities in the intervention programme. Here, we tested whether: (i) baseline house infestation and abundance of *T. infestans* increased across the urban-to-rural gradient, as presumably did the abundance of non-human domestic hosts and refuge availability; (ii) the risk index implemented was able to identify the infested houses; (iii) the association between house infestation and the availability of domestic animals, peridomestic structures and other ecological determinants varied across the gradient; and (iv) urban and peri-urban infestations were spatially aggregated and associated with population settlement history and contact with rural areas.

## Methods

### Study area

**Fig. 1:**
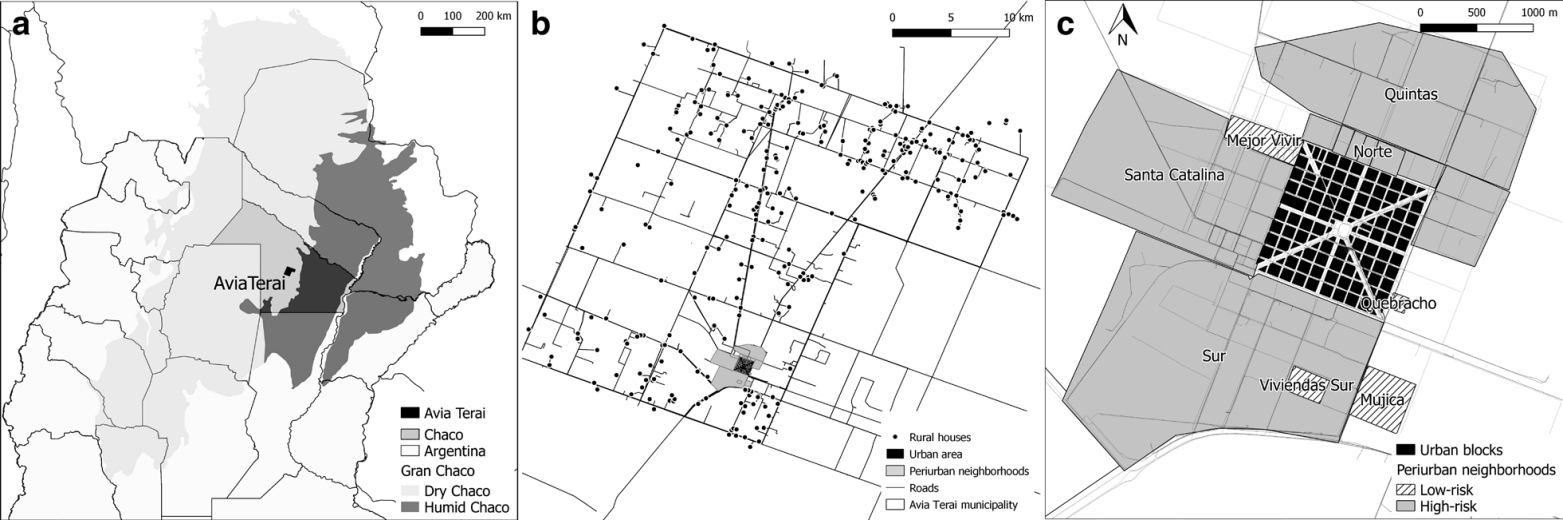
**a** Location of Avia Terai municipality in Chaco, Argentina. **b** Rural, peri-urban and urban areas within Avia Terai municipality. **c** Established peri-urban neighbourhoods (light grey), recent peri-urbanisations (lines) and urban blocks (dark grey), 2015–2016. Black dots in the rural area represent each inhabited or uninhabited house and public building

Avia Terai municipality (26°42′S, 60°44′W), located in Chaco Province, northeast Argentina (Fig. 1), comprised 700 km^2^ inhabited by approximately 13,000 people as of 2016. Three settings were identified mainly based on housing arrangement. The urban section, defined as a place with more than 2000 inhabitants [31], consisted of 100 blocks (of 1 ha each) arranged in a 10-by-10 matrix including 1413 inhabited dwellings. The peri-urban area was highly heterogeneous, with a combination of urbanised blocks and dispersed houses, and included 575 inhabited dwellings grouped in eight neighbourhoods. Four neighbourhoods were recent peri-urbanisations originated from state-funded housing programmes; had similar building characteristics (i.e. absence of peridomestic structures in most houses), and no previous record of triatomine presence. The remaining four peri-urban neighbourhoods (hereafter referred to as peri-urban area) showed suitable conditions for triatomine infestation due to their housing characteristics (i.e. presence of peridomestic structures), and reports of previous infestation. Additional details of the study area are described in Additional file 1: Text S1.

Rural areas comprised 308 inhabited houses as of 2015 (including 34 adjacent houses from neighbouring districts). A typical house compound included one domicile (i.e. an independent structure used as human sleeping quarters), a patio and peridomestic structures, such as kitchens, storerooms, corrals, chicken coops and chicken nests (“nidero”), ovens, among others. Urban or peri-urban houses usually differed from rural houses in the amount of peridomestic structures housing domestic animals (Additional file 2: Figure S1).

Prior to the study, official vector control personnel had conducted a district-wide insecticide spraying campaign to suppress triatomine infestations over 2011–2013. In spite of sizable control efforts, house infestations persisted and local authorities requested additional control interventions, which originated to this study.

### Study design

A cross-sectional survey of house infestation with triatomines using a stratified sampling strategy was conducted across the urban-to-rural gradient between October 2015 and March 2016. All buildings were identified with a unique code. Rural houses were georeferenced using a GPS (Garmin Legend) and identified with a numbered aluminium plate located near the front door. At each inhabited house, we briefly explained the aims of the project to the dwellers and invited them to participate and provide oral consent. Householders were asked if they have sighted any triatomine at their dwellings over the previous year (householders’ notification) after showing them dry specimens of *T. infestans*, *T. sordida* and other Reduviidae, and whether they had peridomestic structures housing animals. These data were combined in a risk index to select the sample of inhabited houses in which triatomine and household surveys were subsequently performed. The index is based on the substantial predictive value of householders’ notifications of house infestations and the widespread occurrence of peridomestic infestations in rural areas [26, 29]. High-risk houses were defined as those that reported triatomine occurrence or had peridomestic structures housing animals; low-risk houses were those that did not meet these criteria. All high-risk houses were inspected for triatomines and surveyed for household attributes; this led to the inclusion of virtually all rural houses and a fraction of urban and peri-urban houses. A systematic sample (30%) of low-risk houses in urban or peri-urban areas was surveyed for triatomines and household attributes using the same procedures. Low-risk houses contiguous to infested houses were additionally inspected for infestation.

### Vector survey

House infestation with triatomines was determined by timed-manual searches (TMS) using a dislodging aerosol (0.2% tetramethrin) (Espacial, Buenos Aires, Argentina). Each domestic and peridomestic structure of the study houses was searched during 15 min by one skilled inspector of the triatomine control programme. Additional methods used to assess house infestation included householders’ bug collections, and triatomine collections during insecticide application (see below). Householders were instructed on how to manipulate the bugs safely and were provided with a labelled self-sealing plastic bag to keep any collected triatomine. All specimens caught were identified taxonomically at the field laboratory [32], counted according to species, stage or sex, and then preserved at − 20 °C as described [28]. Immediately after the baseline survey, vector control personnel sprayed houses with suspension concentrate cypermethrin (Sipertrin, Chemotecnica, Argentina) using a standard (50 mg/m^2^) dose in domiciles and a double dose in peridomiciles. A community-wide spraying was conducted in the rural area, while only infested and adjacent houses were sprayed in urban and peri-urban sections.

A house was considered infested if any live adult or nymph of *T. infestans* was collected by any bug collection method, including TMS, bug collections during insecticide application, and householders’ bug collections [28], unless otherwise noted. Vector abundance was computed as the number of live triatomines collected by TMS per 15 person-min. If a house compound had more than one infested site, the average bug abundance was calculated as the number of live triatomines collected per 15 person-min divided by the number of infested sites. Colonisation was defined as the presence of at least one live nymph of *T. infestans*.

### Sociodemographic survey

A household questionnaire was administered in parallel to triatomine searches. An adult member from every surveyed inhabited house was asked about the number of domestic animals (dogs, cats and poultry) and their resting places, domestic insecticide use, approximate date of the last government-sponsored house spraying with insecticide, number of domestic premises, and number and type of peridomestic structures.

In rural households, we also registered building materials of domestic premises; number of residents; and livestock farming (number of pigs and goats). These additional variables (registered in a subset of peri-urban and urban houses) were recorded over March–June 2017.

### Settlement patterns and triatomine presence

We gathered qualitative data on urban and peri-urban history of settlement and triatomine presence in Avia Terai in urban and peri-urban household surveys and through in-depth interviews. The questionnaires included the timing of household settlement (< 5, 5–15, > 15 years), previous geographical origin of householders (rural, peri-urban, urban or other), whether they had frequent contact with rural areas, and the reasons for such contact (family visit, job or other).

In-depth interviews to 10 residents of urban and peri-urban neighbourhoods were conducted to canvas information about their place of residence, building features, presence of peridomestic structures and triatomines, domestic animal husbandry, and vector control interventions during his/her lifetime. Nine of the interviewees were women (mean age, 53 years-old; age range, 40–72 years-old). Additional interviews to a local writer/historian and a municipal official provided information on population settlement. All the interviews were audio-recorded with the oral consent of the participants.

### Data analysis

House-infestation analyses excluded public buildings and abandoned houses (which had no triatomines except in two occasions) and recent peri-urbanisations (no triatomines ever found). Some rarely infested ecotopes (latrines, trees used by chickens and other ecotopes) were excluded from the number of peridomestic sites.

The sensitivity of the risk index was calculated as the proportion of houses positive by the index (i.e. high-risk houses) among all TMS-positive houses; specificity measured the proportion of houses negative by the index (low-risk houses) among TMS-negative infested houses. The positive and negative predictive values of the index were calculated as the proportion of high-risk houses that were positive by TMS, and the proportion of low-risk houses that were negative by TMS, respectively.

We used the available information to compute a projected prevalence of house infestation for each type of environment or setting (i.e. urban, peri-urban and rural) and house risk level (i.e. high-risk, low-risk and unknown, which included inhabited houses that were closed or refused to participate in the risk assessment survey). The relevant pieces of information were the observed prevalence of house infestation and total number of houses at each setting and risk level. For houses with an unknown risk status, we conservatively assumed they had the same risk distribution and prevalence of infestation as did the surveyed high-risk houses. The projected number of infested houses is the product of the number of houses registered and projected prevalence of infestation at each setting.

The associations between house infestation or bug abundance (outcome variables) and selected explanatory variables with supporting evidence [28] were tested through multiple logistic and negative binomial regression analysis, respectively. Only high-risk dwellings were included in these analyses to avoid perfect separation of data (i.e. all low-risk dwellings were not infested). The explanatory variables included host availability (numbers of human residents, poultry, and dogs or cats), presence of suitable conditions for triatomine colonisation in domiciliary walls (i.e. not fully plastered walls or presence of cracks), reported insecticide application, and type of environment. The numbers of human residents, poultry, and dogs or cats were categorized according to their quartiles. No multicollinearity among explanatory variables was detected (variance inflation factor < 1.5 for every variable). We evaluated the significance of interactions between type of environment and each explanatory variable to determine whether the association varied among environments, and retained in the model those with a significant effect at the 5% level. The overall fit of the logistic model was assessed by the Hosmer–Lemeshow test (pooling the data in 10 groups) and the area under the receiver-operating curve (ROC), where a value of 1 indicates a perfect fit. Statistical analyses were implemented in Stata 14.2 [33].

The spatial distribution of house infestation and bug abundance was assessed through global and local point pattern analyses. The analyses were performed at house level (in the rural area), at block level (in the urban area), and blocks or houses (in the peri-urban neighbourhoods) according to the spatial arrangement of houses. Global spatial analyses of house infestation (rural) and house or block infestation (urban or peri-urban) were performed using the K-function implemented in Programita [34]. Random labelling was used to test the null hypothesis of random events among the fixed spatial distribution of all houses (or blocks). The selected cell size was 300 m for rural houses and 50 m for urban or peri-urban houses or blocks; the maximum distance was set at 15 km and 600 m, respectively (i.e. half of the dimension of the area). Monte Carlo simulations (*n* = 999) were performed and the 95% ‘confidence envelope’ was calculated with the 2.5% upper and lower simulations. Local spatial aggregation of infestation and bug abundance was tested using the Getis statistic (G*) [35] implemented in PPA [36], with the same parameters as for the global analysis.

A contagion index was used to evaluate the existence of spatial aggregation of urban block infestation, considering that the point pattern analysis may be limited by the regular distribution and the number of blocks in the study setting. The contagion index is a landscape metric based on block adjacencies, which describes the probability of two random blocks having at least one infested house [37]. This metric is affected both by the proportion of infested blocks and their interspersion. It tends to 0 if infested blocks are unevenly distributed, and 1 if all blocks have the same infestation status (i.e. all infested or all uninfested). In order to compute the contagion index, we turned the urban blocks into a matrix and used the *landscapemetrics* package in R to estimate the index [38]. We estimated the probability of the observed contagion index given its expected distribution in simulated landscapes having the same infestation prevalence and number of blocks. We ran 10,000 random permutations of the matrix and calculated the contagion index in each run to estimate the parameters of a normal distribution of the contagion index within the range of contagion values considered (determined by the large number of permutations). The full reproducible code has been made publicly available [39].

## Results

### Triatomine infestation

The house infestation risk index was assessed in 1050 (74.3%) urban houses, 253 (82.4%) houses in established peri-urban neighbourhoods, 165 (61.6%) from recent peri-urbanisations, and 276 (89.6%) rural dwellings. Houses whose risk index was not assessed were closed (22%) or their dwellers refused to participate in the study (2%). In total, 972 inhabited houses were inspected by TMS (Table 1). All TMS-positive dwellings (or by any bug collection methods) were high-risk houses, whereas every low-risk house inspected was TMS-negative across the gradient. The sensitivity and negative predictive value of the index were 100% across settings. Specificity was higher in urban (39.3%) or peri-urban (39.9%) settings than in rural (14.5%) ones. The positive predictive value was 17.3–46.3% across the gradient.

**Table 1:** Distribution of house infestation and abundance of *Triatoma infestans* according to type of environment, Avia Terai, 2015–2016

Environment	No. of dwellings registered	No. of dwellings not inspected		% dwellings infested by TMS (No. inspected)		Projected infestation (No. of infested dwellings)	Median bug abundance (IQR)
		Closed/refused	Low-risk	Low-risk houses^a^	High-risk houses^a^		
Rural	308	16/16	0	0.0 (23)	46.3(253)	42.4 (131)	11 (5–23)
Peri-urban							
Established	307	44/10	43	0.0 (61)	38.3 (149)	22.5 (69)	7 (3–18)
Recent urbanisations	268	102/1	95	0.0 (45)	0.0 (25)	0.0 (0)	–
Urban	1413	346/17	634	0.0 (145)	17.3 (271)	4.5 (63)	8 (2–16)
Total	2296	508/44	772	0.0 (274)	31.7 (698)	12.7 (291)^b^	10 (4–19)

^a^High-risk houses: those where householders notified the presence of *T. infestans* or the existence of peridomestic structures. Low-risk houses did not meet these criteria

^b^Unweighted total

House infestation, as determined by TMS in high-risk dwellings, significantly increased from 17.3% to 46.3% across the urban-to-rural gradient (*χ*^2^ test, *df* = 2, *P* < 0.001) (Table 1). These indices rose by < 2% when the combined result of all bug collection methods was considered. The median abundance of *T. infestans* per infested house was higher in rural areas (median: 11, interquartile range, IQR: 5–23) than in peri-urban (median: 7, IQR: 3–18) and urban (median: 8, IQR: 2–16) settings (negative binomial regression, peri-urban: RA = 0.87, 95% CI: 0.63–1.20; urban: RA = 0.66, 95% CI: 0.46–0.93). In total, 640, 1560 and 3391 *T. infestans* were caught across the urban-to-rural gradient. The projected prevalence of house infestation increased from 4.5%, 22.5% to 42.4% across the urban-to-rural gradient, respectively, including an estimated number of 63, 69 and 131 infested houses, respectively (Table 1).

Domiciliary infestation increased from 1.1% (urban), 8.7% (peri-urban) to 9.9% (rural) in high-risk dwellings inspected by TMS (Fisher’s exact test, *df* = 2, *P* < 0.001). Colonisation occurred in 88.5%, 85.0% and 90.8% of the infested houses across the urban-to-rural gradient, respectively. The prevalence of house infestation was highly heterogeneous (range, 17–63%) in established peri-urban neighbourhoods, and nil in recent peri-urbanisations.

The infestation prevalence derived from householders’ notifications of *T. infestans* was significantly higher than that estimated by the combined results of all collection methods both for the entire municipality and for each separate environment (exact McNemar’s test, *df* = 1, *P* <  0.001 in all cases). The relative odds of house infestation was 2.9–18.1 times higher in dwellings where householders notified *T. infestans* than in those that did not (overall: OR: 6.9, 95% CI: 4.8–9.9; urban: OR: 2.9, 95% CI: 1.6–5.4; peri-urban: OR: 18.1, 95% CI: 6.8–48.1; rural: OR: 5.5, 95% CI: 3.0–10.0).

**Fig. 2:**
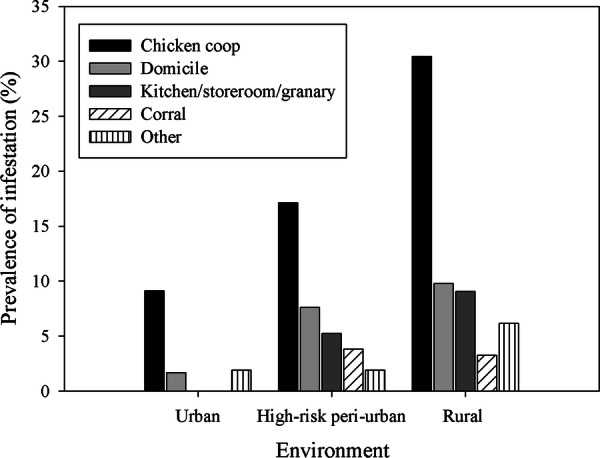
Distribution of house infestation with *T. infestans* (as determined by the combined outcome of all bug collection methods) by ecotope according to the type of environment, Avia Terai, 2015–2016

The ecotope-specific prevalence of house infestation peaked in peridomestic structures housing chickens in urban (9.1%), peri-urban (17.1%) and rural (30.4%) settings (Fig. 2). Ecotopes housing chickens concentrated 60–73% of all houses with *T. infestans* as revealed by the combined result of all bug collection methods. Moderate infestation rates were revealed in kitchens, storerooms and granaries in rural (9.1%) and peri-urban (5.2%) areas, and in goat and pig corrals (3.8%) in peri-urban houses; these ecotopes accounted for 13–21% of infested houses. Dog resting places and stacked materials (‘other’) accounted for 15.4% of all infested urban houses. No significant association was found between domestic and peridomestic infestation stratified by type of environment (Cochran–Mantel–Haenszel *χ*^2^ test, *df* = 2, *P* = 0.7).

*Triatoma sordida* was collected by TMS in 0.0%, 1.0% and 7.3% of the inspected houses across the urban-to-rural gradient, respectively, and almost exclusively occurred in peridomestic structures occupied by chickens. One *P. geniculatus* adult male was collected in each of two rural domiciles.

### Spatial analysis

**Fig. 3:**
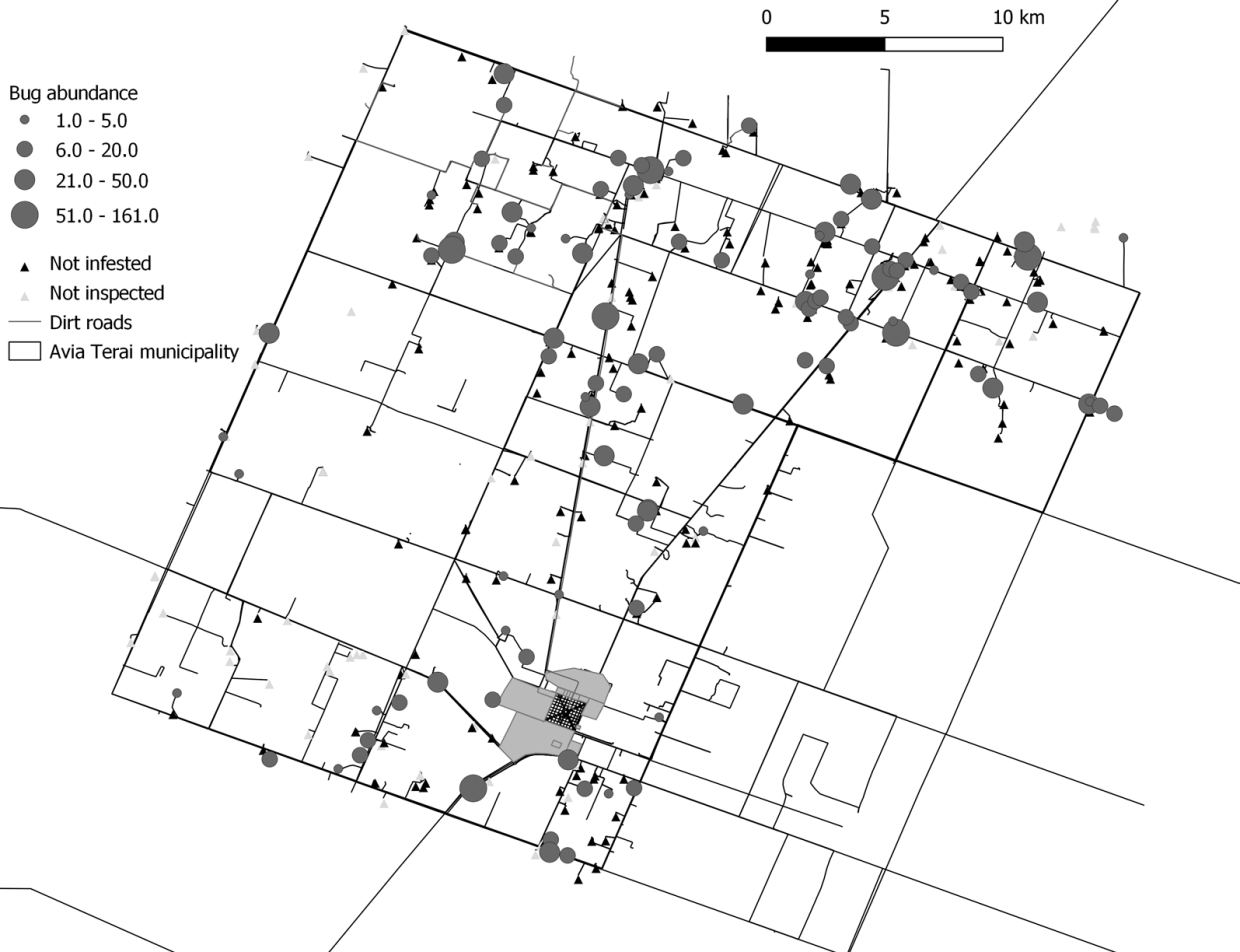
Spatial distribution of the house abundance of *T. infestans* in the rural environment, Avia Terai, 2015. ‘Not inspected’ included inhabited houses that were closed or households that refused to participate, uninhabited houses and public buildings. Urban (dark grey) and peri-urban (light grey) infestation not shown

**Fig. 4:**
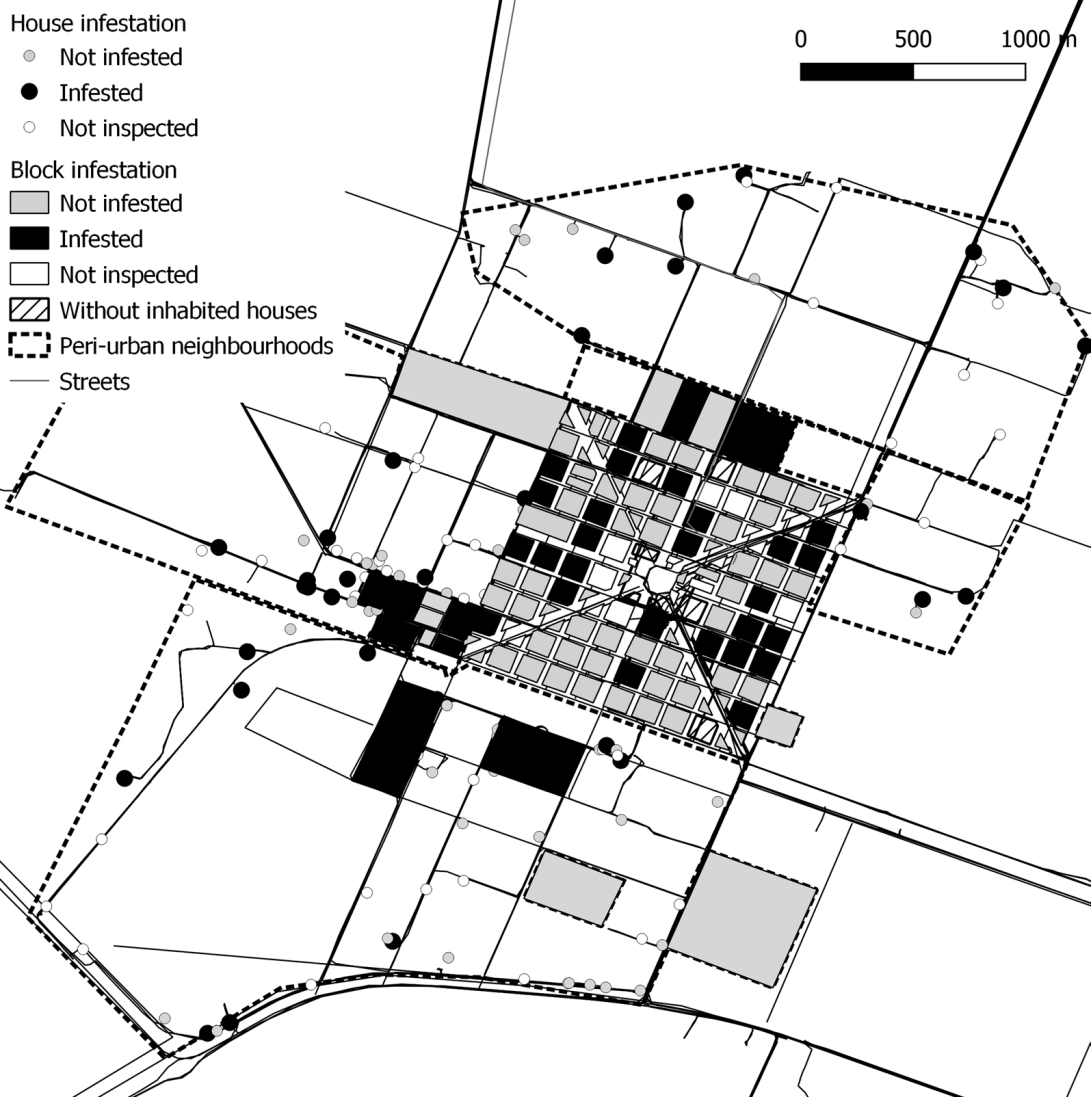
Spatial distribution of house infestation with *T. infestans* in peri-urban and urban environments at house and block level, Avia Terai, 2015–2016

No global or local spatial aggregation of infestation was detected at the house level in the rural environment, or at the house/block level in urban and peri-urban areas (Figs. 3, 4). Local spatial analyses of bug abundance identified one cluster including three infested houses in one peri-urban neighbourhood (Santa Catalina) and one cold spot in another (Barrio Sur) (Fig. 4). The estimated probability of the observed contagion index given the number and spatial distribution of infested blocks (Pb of observed contagion = 0.68) was greater than the alpha value considered (0.05), indicating that it was not significantly aggregated, in agreement with the outcome of point pattern analysis.

### Household characteristics

The median number of inhabitants per dwelling was significantly lower (Kruskal-Wallis test, *χ*^2^ = 22.53, *df* = 3, *P* < 0.001) in rural areas (3) than in peri-urban (4) and urban (4) areas (Additional file 3: Table S1). By contrast, the household presence of dogs, cats, poultry, pigs and goats significantly increased across the urban-rural gradient (*χ*^2^ = 17.91–201.10, *df* = 2, *P* < 0.001, for each type of host separately) (Table 2), as did their abundance (Kruskal–Wallis tests, *χ*^2^ = 71.60–261.83, *df* = 3, *P* < 0.001 in all cases) (Additional file 3: Table S1). Pigs and goats were raised in 35% and 6% of peri-urban houses, respectively; both hosts also occurred in urban houses (< 7%). The presence of peridomestic structures steadily increased from 55% in urban houses, 70% in peri-urban neighbourhoods, to 91% in rural houses (*χ*^2^ = 101.80, *df* = 2, *P* < 0.001). The occurrence of domestic animals and of peridomestic structures was minimal in recent peri-urbanisations except for dogs (Table 2). The occurrence of poultry was significantly and positively associated with the presence of peridomestic structures (Cochran–Mantel–Haenszel test, *df* = 1, *P* < 0.001), with no evidence of heterogeneity among environments (Cochran–Mantel–Haenszel test, *df* = 3, *P* = 0.5). Domiciliary walls had suitable conditions for *T. infestans* in 26%, 40% and 65% of urban, peri-urban and rural houses, respectively.

**Table 2:** Distribution of housing and socio-demographic characteristics by type of environment, Avia Terai, Chaco, 2015–2016

Attribute	% of households with the attribute (No. surveyed)			
	Rural	Peri-urban		Urban
		Established neighbourhoods	Recent urbanisations	
Housing characteristics				
Domiciles with suitable walls	64.5 (273)	40.1 (147)^a^	0.0 (31)^a^	25.6 (305)^a^
Domiciles with suitable roofs	22.9 (271)	22.5 (147)^a^	3.2 (31)^a^	17.7 (305)^a^
Peridomestic structures	91.2 (274)	69.9 (199)	22.7 (66)	54.7 (386)
Household size (≥ 4 residents)	46.9 (273)	59.6 (151)^a^	83.9 (31)^a^	63.3 (305)^a^
Insecticide applications				
Insecticide use	81.3 (267)	69.8 (189)	71.0 (62)	76.6 (364)
Pyrethroid use	31.8 (267)	6.4 (189)	3.2 (62)	12.9 (365)
≤ 4 years since last spraying	81.5 (135)	55.1 (136)	20.0 (50)	51.2 (248)
Presence of domestic animals				
Dogs	97.0 (266)	90.7 (204)^a^	77.3 (66)^a^	87.5 (409)^a^
Cats	69.4 (242)	47.3 (201)^a^	21.2 (66)^a^	40.3 (409)^a^
Poultry	91.0 (266)	53.9 (193)^a^	22.7 (66)^a^	48.0 (406)^a^
Pigs	62.4 (258)	35.4 (147)^a^	0.0 (31)^a^	6.2 (305)^a^
Goats	40.7 (258)	6.1 (147)^a^	0.0 (31)^a^	1.0 (305)^a^

^a^Based on data registered in 2016–2017 surveys

Domestic insecticide use was reported in > 70% of households across the municipality (Table 2), with low-concentration aerosols being the most frequent type (> 68%). Pyrethroid applications were significantly more frequent in rural houses (31.8%) than in other settings (6–13%) (established peri-urban: OR: 0.15, 95% CI: 0.07–0.28; recent peri-urban: OR: 0.07, 95% CI: 0.02–0.31; urban: OR: 0.32, 95% CI: 0.21–0.48, with rural houses as the reference category) (Table 2). Householders reported that over the previous four years, official vector control personnel had sprayed insecticide at their premises at least once in 51.2%, 55.1% and 81.5% of urban, peri-urban and rural houses, respectively.

Most households from peri-urban neighbourhoods reported long-term residence (> 15 years); previous residence in an uninfested urban house, and no frequent contact with rural areas (Additional file 4: Table S2). In contrast, most households in recent peri-urbanisations had settled in over the previous five years (*χ*^2^ = 54.49, *df* = 2, *P* < 0.001), and had more frequent contact with rural areas than peri-urban households (*χ*^2^ = 10.50, *df* = 1, *P* = 0.001). Compared to the latter, urban households reported a higher frequency of long-term residence (*χ*^2^ = 12.42, *df* = 2, *P* = 0.002), and more frequent contact with rural areas (*χ*^2^ = 14.50, *df* = 1, *P* < 0.001).

### Determinants of house infestation and bug abundance

Multiple logistic regression analysis showed that overall house infestation was significantly and positively associated with the household number of poultry and the presence of suitable conditions for *T. infestans* in domiciliary walls, and was negatively associated with domestic insecticide use (Table 3). Peri-urban house infestation almost doubled that in the urban setting, and was not significantly associated with the household number of human residents and of dogs or cats. A significant two-way interaction was found between the type of environment and number of poultry, and between the type of environment and number of residents: the effects of having *>* 25 poultry were 15 times higher in peri-urban houses than in urban houses (OR: 15.03, 95% CI: 1.66–136.21), and the effects of having > 5 residents were 4 times higher in rural than in urban houses (OR: 4.19, 95% CI: 1.31–13.37). The Wald test was significant in all cases (*P* < 0.001). The logistic model for infestation (Hosmer–Lemeshow test, *χ*^2^ = 5.22; *df* = 8; *P* = 0.73) and the area under the ROC curve (0.76) indicated a good fit.

**Table 3:** Odds ratio (OR) and relative abundance (RA) for each predictor of house infestation and abundance of *Triatoma infestans* in high-risk houses across the municipality of Avia Terai (*n* = 518 houses)

Variable	House infestation		Bug abundance	
	OR	95% CI	RA	95% CI
Domestic insecticide use				
No	1.00	–	1.00	–
Yes	0.57	0.34–0.95*	0.58	0.32–1.07
No. of residents				
1–2	1.00	–	1.00	–
3–4	0.94	0.56–1.58	0.94	0.51–1.74
> 5	0.70	0.43–1.14	0.47	0.26–0.85*
No. of dogs or cats				
0	1.00	–	1.00	–
1–2	1.95	0.30–12.55	3.50	0.55–22.19
˃ 2	1.48	0.26–8.32	3.66	0.70–19.03
No. of poultry				
0	1.00	–	1.00	–
1–5	1.29	0.46–3.68	1.61	0.60–4.34
6–25	5.59	2.58–12.11*	6.13	3.01–12.50*
˃ 25	12.72	5.64–28.70*	13.31	6.41–27.64*
Suitable walls				
No	1.00	–	1.00	–
Yes	1.61	1.05–2.45*	1.21	0.72–2.02
Environment				
Urban	1.00	–	1.00	–
Peri-urban	1.74	0.96–3.14	1.06	0.52–2.18
Rural	1.29	0.75–2.20	1.80	0.97–3.36

*P < 0.05

Negative binomial regression analysis showed that bug abundance was significantly associated with the household number of poultry (positively) and of human residents (negatively). The predicted relative bug abundance in rural houses almost doubled that in urban houses (Table 3). A significant interaction was found between the type of environment and number of poultry: the effects of > 25 poultry were almost 10 times higher in peri-urban compared to urban houses (OR: 9.74, 95% CI: 1.03–92.39). No significant associations were found between bug abundance and insecticide use, the household number of dogs or cats, and the interaction between type of environment and number of residents.

In the urban setting, house infestation was positively associated with duration of residence, and was significantly lower if the former place of residence was urban compared to those who had resided in rural areas. Peri-urban house infestation was not associated with any settlement-related variable (Table 4).

**Table 4:** Distribution of house infestation with *Triatoma infestans* according to variables associated with settlement and contact with rural areas in peri-urban and urban settings

Variable	Urban			Peri-urban		
	*n*	OR	95% CI	*n*	OR	95% CI
Duration of residence (years)	375			167		
< 5		1.0			1.0	
5–15		9.4	1.2–76.3*		1.1	0.4–2.7
> 15		14.3	1.8–112.2*		0.6	0.3–1.5
Former place of residence	325			117		
Rural		1.0			1.0	
Peri-urban		–	–		1.4	0.4–4.8
Urban		0.4	0.2–0.9*		0.8	0.3–2.3
Other		0.6	0.2–2.2		0.5	0.1–5.1
*T. infestans* in former residence	307			98		
No		1.0			1.0	–
Yes		1.2	0.6–2.3		1.2	0.5–3.0
Contact with rural areas	343			123		
No		1.0			1.0	
Yes		1.0	0.5–1.8		0.7	0.3–1.8

*P < 0.05

In-depth interviews traced house infestation with triatomines back to the 1970s both in urban and peri-urban areas, and at least to the 1960s in the rural environment. The people who were interviewed (aged 40–72 years-old) agreed on the frequent finding of triatomines in chicken coops and inside domiciles of urban and peri-urban houses since a young age. A fragment of an interview to a 50-year-old woman, a lifetime resident in the urban area, described the past as follows: “I remember when I was at school, I was 9–10 years-old, the house of a neighbour, in the center of town, was full of *vinchucas* (triatomines). The teacher asked us to take *vinchucas* to the school and I brought a full jar collected in that house. In that time, they were everywhere, inside the house, in the walls, beneath the mattresses, in the chicken coops, everywhere. Now is different.”

## Discussion

Our study discloses the sizable dimensions of urban and peri-urban house infestation with triatomines in comparison with rural areas. Their relative importance can be viewed from the perspective of the proportion of infested houses (prevalence) or from the number of infested houses that require treatment at the aggregate (setting) level. While the projected prevalence in urban (4.5%) and rural (42.4%) settings were one order of magnitude apart, the combined number of infested houses in peri-urban and urban areas equalled that number in rural areas (132 *versus* 131, respectively) because of vast differences in the relative number of houses (5.8×). Average bug abundance was greater in rural areas, but when the total collected is considered at the aggregate level, the relative magnitude of urban or peri-urban *versus* rural setting was more balanced.

The rapid screening of house infestation risk allowed identification of all houses that subsequently proved to be TMS-positive, and correctly classified as low-risk dwellings according to TMS-based outcomes. Although the absence of a gold standard method to assess house infestation with triatomines hinders the evaluation of any risk index, we obtained similar results when the combined outcome of several bug detection methods was considered. In general, risk stratification scores that predict the probability of house infestation with triatomines are very much needed [40]. In Bolivia, a risk score showed high sensitivity and specificity when householders’ reports of house infestation were used as the outcome [27]. Recently, a multivariate index of social vulnerability identified households at higher risk of *T. cruzi* transmission in indigenous communities of the Argentine Chaco [30].

The risk index, herein implemented in urban and peri-urban settings, derived from the fact that householder detection of triatomines in domestic premises performed better than other methods in multiple rural locations, especially at low triatomine densities [29, 40]. Furthermore, the presence of peridomestic structures housing domestic animals synthesises the combined effects of refuge and host availability on site infestation, and was especially relevant because triatomines prevailed in peridomestic structures. Both variables can be easily registered by primary healthcare agents or local residents as part of a community-based vector surveillance.

Both house infestation and bug abundance across Avia Terai were much higher than expected considering the few years which had elapsed since the last insecticide spraying campaign, with > 40% of rural houses infested for reasons that remain unclear. Nevertheless, the observed infestation levels underestimate the true prevalence levels because TMS has limited sensitivity, especially at low triatomine densities [28, 41]. In other endemic rural areas of the Argentine Chaco, such high levels of house infestation were hit after a decade during which no insecticide spraying or effective vector surveillance were implemented [25, 26, 28, 42, 43]. Peri-urban infestation rates in Avia Terai were much higher than in a recently-established peri-urban slum in Chaco Province [23], and were similar to peridomestic infestation levels (30–50%) in peri-urban settlements of Arequipa, Peru [9, 22].

Our study revealed an increasing urban-to-rural gradient of house infestation and abundance of *T. infestans*. House infestation prevalence increased by an order of magnitude across the gradient, in parallel to an increasing trend in the abundance of domestic animals and of peridomestic structures. Triatomine populations were largely associated with the household number of poultry across settings, supporting that chickens and chicken nests were key hosts and habitats, respectively [25, 26, 44]. Structures housing chickens accounted for 60–73% of all infested houses detected across the municipality. The key role of peridomiciles was evident both in peri-urban and urban areas, and adds to its well-known relevance in rural settings (e.g. [43, 45]). Guinea pigs [9] and pigeons [10, 46] played analogous key roles in other urban and peri-urban landscapes. A large fraction of urban and peri-urban houses in Avia Terai raised poultry or other domestic animals (mainly pigs or goats) in conditions suitable for triatomines. House infestation was not significantly associated with the type of environment using multiple regression analysis, suggesting that variations between environments were captured by other explanatory variables such as the number of poultry in a household. The lack of a strict association between triatomine infestations and rural settings was clearly stated by Mayer & Alcaraz in 1954 [47]: “The predominance of Chagas disease in rural areas is more apparent than real, since it is not determined by the environment but associated with the special conditions that deficit human dwellings usually meet (…)”.

Domiciliary infestations pose potential risks of *T. cruzi* transmission to humans; levels ranged from 1.1 to 9.9%, and accounted for 14–27% of infested houses across settings. Infestation levels were most likely associated with the predominant building characteristics of domiciles, most of them providing few refuges to the vector. While > 60–75% of urban and peri-urban domiciles had plastered walls and tin roofs, almost 65% of rural dwellings had unplastered or partially plastered brick-and-cement walls and tin roofs. Low domiciliary infestation levels were also related to domestic insecticide use and the higher efficacy of pyrethroids indoors [45], either because of the prior spraying campaign and/or householders’ applications. These results reinforce that housing quality and householder attitudes and practices exert important effects on house infestation with triatomines [28].

Several pieces of evidence support that current urban or peri-urban infestations were mostly independent of, or had weak links to, rural areas or products: (i) urban or peri-urban foci display high colonisation indices suggesting they did not represent recent bug invasions from external foci; (ii) house infestation was not spatially aggregated, as expected from bug spread from a few recently-established foci; (iii) long-established urban houses had a 5–8 times higher relative odds of being infested than recently-established urban houses; (iv) peri-urban neighbourhoods with higher infestation prevalence were among the oldest, and recent urbanisations were triatomine-free, as expected from their improved building features and insufficient exposure time; and (v) no significant association was found between urban or peri-urban infestation and having former residency in rural areas or frequent contact with them. Additionally, peri-urban and urban infestations in Avia Terai were traced back at least to the 1970s. Long-time residents believed that current infestation levels were markedly inferior to those existing decades before. The available evidence supports that *T. infestans* is a long-established occupant of urban, peri-urban and rural settings in Avia Terai.

Although the published reports of urban and peri-urban foci of *T. infestans* and other triatomine species have apparently increased during the last decades (see “Background”), the occurrence of urban house infestations is long standing. Many cities in Argentina displayed high prevalence rates of house infestation with *T. infestans* and bug infection in the 1940s [48–50], and this also occurred with other triatomines across Latin American cities (e.g., [51]). The Argentine Chagas vector control programme stated in 1977: “We have found *T. infestans* in every corner where a human dwelling exists” [50]. Vector-borne transmission was well-established in peri-urban neighbourhoods of Resistencia (Chaco Province) over the 1950s, with high levels of house infestation (40%) and *T. cruzi* infection in bugs, dogs or cats and children [47, 49]. Therefore, it seems that urban and peri-urban infestations have been overlooked, underrated or not addressed effectively and do not represent an emerging issue.

We identified additional limitations not discussed above: (i) although several sociodemographic variables were registered one year after triatomine searches, their rate of change is usually slow and the lag would eventually introduce minor changes over such a short time span; (ii) inferences from in-depth interviews are limited by the reduced number of people interviewed, their age, gender and origin, and therefore represent a preliminary assessment of local settlement patterns; (iii) whether the false-negative rate varies with housing quality or type of setting is unknown, and may affect the relative magnitude of house infestation between settings; (iv) the projected prevalence was estimated assuming that closed/refused houses had the same risk index distribution and infestation prevalence than surveyed houses; and (v) at low vector abundance, the risk index could misclassify a high-risk house if the person interviewed was unaware of bug observations of other household residents, or by a recent infestation of any peridomestic ecotope with which they have little contact. The distribution of *T. cruzi* infection and its relation to host bloodmeal sources by type of setting will be treated separately.

## Conclusions

Our study has implications for improved vector control. Triatomine control operations in rural areas traditionally follow the rules of continuity (in time) and contiguity (in space), and seek to achieve full coverage of house units depending on village-level house infestation rates. An epidemiological scenario where high infestation prevalence in rural houses coexists with extended low-density infestations in urban and peri-urban areas presents new challenges to typically resource-constrained triatomine control programmes. Although the proximity between houses reduces travel times and increases productivity relative to sparsely populated rural areas, short inter-house distances facilitate the spread of bugs. Our simple stratification index allowed the identification of high-risk houses and substantial savings in labour and other resources for prompt action. For example, the estimated prevalence of house infestation in the urban setting (4.5%) was close to the threshold (5%) used to define whether all houses in a rural village or district should be sprayed with insecticide. Achieving a high degree of coverage of house infestation assessment or insecticide treatment is virtually impractical or unfeasible in densely populated urban areas. Therefore, vector control tactics adapted to urban and peri-urban settings need to be developed and tested. Another relevant issue relied on the fact that the generalized practice of raising poultry and the associated peridomestic structures were the main factors associated with house infestation and bug abundance across the municipality. They are ideal targets for an integrated vector management strategy including: good husbandry practices that improve animal health and avert other threats (e.g. avian influenza, attraction and blood-feeding of sand flies); designing appropriate chicken coops less susceptible to infestation; and manually removing triatomines on a periodic basis. These actions would bring additional savings associated with recurrent insecticide applications.

## Supplementary information

**Additional file 1: Text S1.** Detailed description of the study area.

**Additional file 2: Figure S1.** Typical houses and peridomestic structures of Avia Terai. **a** Urban houses and peridomestic structures housing chickens. **b** A typical house of an established peri-urban neighbourhood and peridomestic structures associated with chickens and a pig corral. **c** A rural house and its associated peridomestic structures housing chickens and pigs.

**Additional file 3: Table S1.** Household number of inhabitants, domestic animals and peridomestic structures by type of environment, Avia Terai, Chaco.

**Additional file 4: Table S2.** Summary of settlement characteristics by type of environment, Avia Terai, Chaco, 2015–2016. *Abbreviation*: NR, data not registered.

**Additional file 5: Table S3.** Individual data including house infestation, environmental and demographic variables, Avia Terai, Chaco, 2015–2016.

## Data Availability

Data supporting the conclusions of this article are included within the article and its additional files. The datasets generated during and/or analysed during the present study are available in Additional file 5: Table S3.

## Abbreviation

TMS: timed-manual searches
