# Supplementary material for: Urbanisation, risk stratification and house infestation with a major vector of Chagas disease in an endemic municipality of the Argentine Chaco
Source: Parasit Vectors. 2020 Jun 18;13:316. doi: 10.1186/s13071-020-04182-3 (PMC7302373; doi:10.1186/s13071-020-04182-3)
Supplement: Supplementary file 1 — Additional file 1: Text S1. Detailed description of the study area. [file 13071_2020_4182_MOESM1_ESM.docx]

**Additional file 1: Text S1**

**Study area**

The population mainly includes descendants of immigrants from neighbouring provinces and countries, and from Europe (i.e., creoles). According to local referents, the first immigrants arrived in 1912 following the construction of the railway; the town was officially founded in 1921. The area was occupied by nomadic indigenous communities that did not settle in permanently because of the scarcity of water sources. The local economy is based on forest activities, agriculture (soybean, sunflower, corn, cotton) and livestock farming (mainly goats, pigs and cows). The climate is sub-tropical and the landscape is dominated by agricultural fields including small forest fragments.

The urban area included two primary schools and one secondary school, two kindergartens, and one basic hospital as of 2016. The town adopted an urban layout over the 1990s following several government-sponsored housing initiatives. No school or healthcare post were present in peri-urban neighbourhoods as of 2016, and most households lacked land titles. Two of them were the first to be occupied on the early 20^th^ century. All peri-urban and urban neighbourhoods had similar access to water, electricity, and waste disposal service. Rural areas included eight primary schools and a secondary school, and two healthcare posts.

A total of 1,050 inhabitants were registered in the rural area at baseline (October 2015). According to local municipal authorities, approximately 12,000 people resided in urban or peri-urban sections of the municipality.
