# Supplementary figures and images for: Urbanisation, risk stratification and house infestation with a major vector of Chagas disease in an endemic municipality of the Argentine Chaco
Source: Parasit Vectors. 2020 Jun 18;13:316. doi: 10.1186/s13071-020-04182-3 (PMC7302373; doi:10.1186/s13071-020-04182-3)

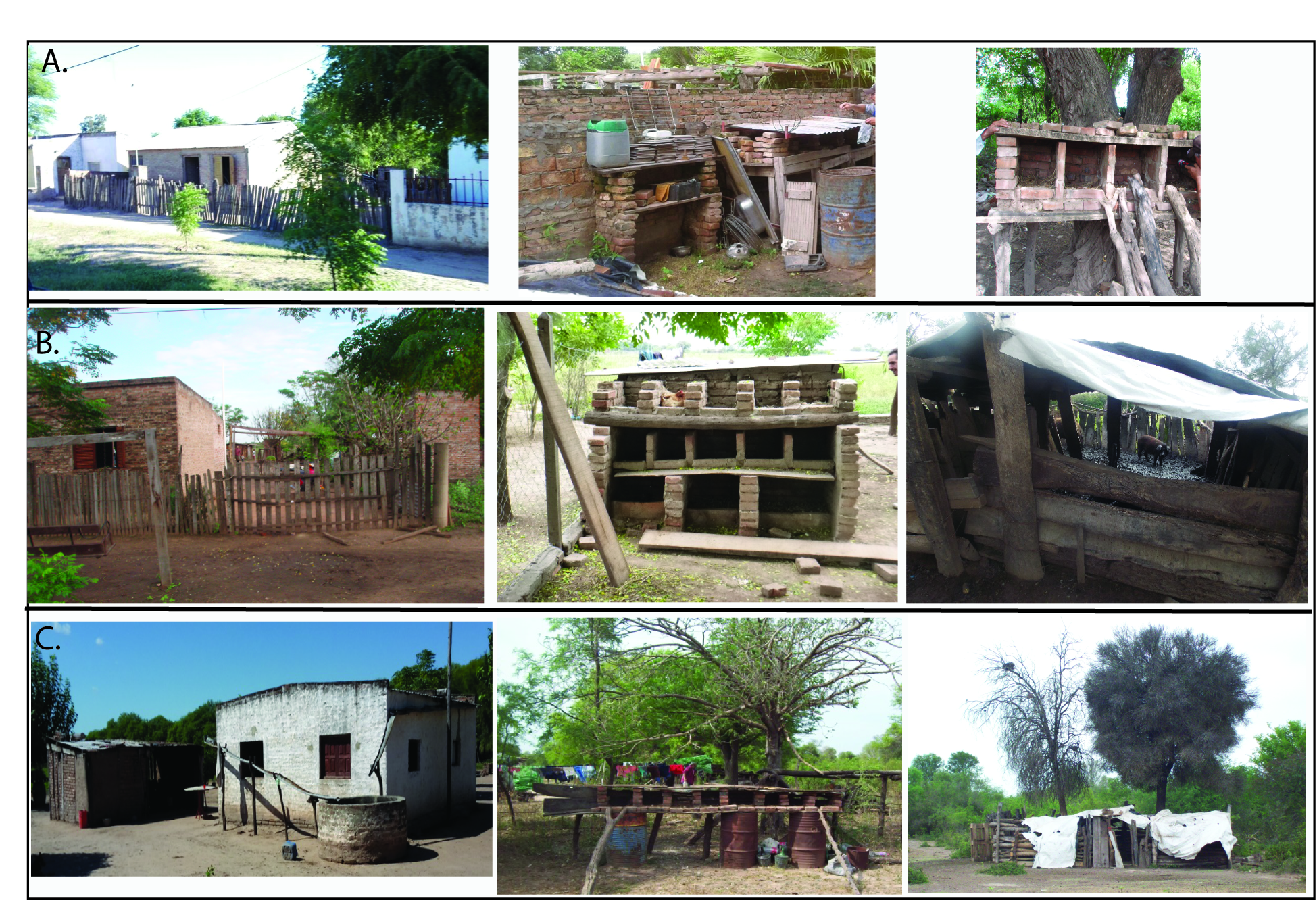

Supplement: Supplementary file 2 — Additional file 2: Figure S1. Typical houses and peridomestic structures of Avia Terai. a Urban houses and peridomestic structures housing chickens. b A typical house of an established peri-urban neighbourhood and peridomestic structures associated with chickens and a pig corral. c A rural house and its associated peridomestic structures housing chickens and pigs. [file 13071_2020_4182_MOESM2_ESM.tif]
