# Supplementary material for: Urbanisation, risk stratification and house infestation with a major vector of Chagas disease in an endemic municipality of the Argentine Chaco
Source: Parasit Vectors. 2020 Jun 18;13:316. doi: 10.1186/s13071-020-04182-3 (PMC7302373; doi:10.1186/s13071-020-04182-3)
Supplement: Supplementary file 3 — Additional file 3: Table S1. Household number of inhabitants, domestic animals and peridomestic structures by type of environment, Avia Terai, Chaco. [file 13071_2020_4182_MOESM3_ESM.docx]

**Additional file 3: Table S1.** Household number of inhabitants, domestic animals and peridomestic structures by type of environment, Avia Terai, Chaco.

| **Host** | **Median (Q1-Q3); No. of households** | | | |
| --- | --- | --- | --- | --- |
|  | **Rural** | **Peri-urban neighbourhoods** | **Recent urbanisations** | **Urban** |
| Humans | 3 (2–5); 273 | 4 (2–6); 151 | 6 (4–7); 31 | 4 (3–5); 305 |
| Dogs | 3 (2–5); 266 | 3 (1–4); 204 | 1 (1–3); 66 | 2 (1–3); 409 |
| Cats | 1 (0–2); 242 | 0 (0–1); 201 | 0 (0–0); 66 | 0 (0–1); 409 |
| Poultry | 30 (12–50); 266 | 3 (0–20); 193 | 0 (0–0); 66 | 0 (0–10); 406 |
| Pigs | 3 (0–10); 239 | 0 (0–2); 147 | 0 (0–0); 31 | 0 (0–0); 305 |
| Goats | 0 (0–10); 239 | 0 (0–0); 147 | 0 (0–0); 31 | 0 (0–0); 305 |
| Peridomestic structures | 3 (2–4); 274 | 1 (0–3); 199 | 0 (0–0);66 | 1 (0–1); 386 |
