# Supplementary material for: Urbanisation, risk stratification and house infestation with a major vector of Chagas disease in an endemic municipality of the Argentine Chaco
Source: Parasit Vectors. 2020 Jun 18;13:316. doi: 10.1186/s13071-020-04182-3 (PMC7302373; doi:10.1186/s13071-020-04182-3)
Supplement: Supplementary file 4 — Additional file 4: Table S2. Summary of settlement characteristics by type of environment, Avia Terai, Chaco, 2015–2016. Abbreviation: NR, data not registered. [file 13071_2020_4182_MOESM4_ESM.docx]

**Additional file 4: Table S2.** Summary of settlement characteristics by type of environment, Avia Terai, Chaco, 2015–2016. *Abbreviation*: NR, data not registered.

|  | **% of households with the feature (no. surveyed)** | | | |
| --- | --- | --- | --- | --- |
| **Settlement features** | **Rural** | **Established peri-urban** | **Recent peri-urbanisation** | **Urban** |
| Duration of residence (years) |  |  |  |  |
| < 5 | NR | 32.2 (171) | 79.7 (69) | 18.6 (382) |
| > 15 | NR | 43.3 (171) | 0.0 (69) | 51.1 (382) |
| Previous residence |  |  |  |  |
| rural area | NR | 23.7 (118) | 20.7 (58) | 42.0 (333) |
| urban area | NR | 49.2 (118) | 56.9 (58) | 46.0 (333) |
| Triatomines in previous residence | NR | 40.4 (99) | 46.4 (56) | 51.4 (313) |
| Frequent contact with rural areas | NR | 30.4 (125) | 58.1 (43) | 50.1 (349) |
